# Three‐dimensional optically cleared tissue imaging for analyzing endoscopic images of gastrointestinal neoplasms (with video)

**DOI:** 10.1111/den.15000

**Published:** 2025-02-03

**Authors:** Koki Nakamura, Koki Morishita, Nobuhiko Onda, Ikuko Sakai, Shinya Matsumoto, Eri Tamura, Yuta Kouyama, Yushi Ogawa, Masashi Misawa, Takemasa Hayashi, Hideyuki Miyachi, Shin‐ei Kudo, Tetsuo Nemoto

**Affiliations:** ^1^ Department of Biological Evaluation Analysis Technology Olympus Medical Systems Corp. Tokyo Japan; ^2^ Department of Optical Engineering Olympus Medical Systems Corp. Tokyo Japan; ^3^ Digestive Disease Center Showa University Northern Yokohama Hospital Kanagawa Japan; ^4^ Department of Diagnostic Pathology and Laboratory Medicine Showa University Northern Yokohama Hospital Kanagawa Japan

**Keywords:** 3D imaging, endoscopy, gastrointestinal neoplasm, gastrointestinal tract, narrow band imaging

## Abstract

**Objectives:**

To develop a procedure that matches magnifying endoscopic images with narrow‐band imaging to 3D tissue structures using a tissue‐clearing technique and to qualitatively and quantitatively analyze specified structures in gastrointestinal neoplasms.

**Methods:**

Endoscopically resected formalin‐fixed paraffin‐embedded gastrointestinal tissues (three esophagus, four stomach, seven colon) were made transparent by ethyl cinnamate. They were then subjected to fluorescent staining of nuclei and blood vessels followed by 3D imaging using a confocal laser scanning microscope. A one‐to‐one correspondence between magnifying endoscopic and 3D reconstructed images was established using vessels and crypts with characteristic shapes as guides, and the depth and caliber of specified vessels were measured.

**Results:**

All tissues were optically cleared, which allowed 3D visualization of vascular structures and nuclei in all layers. In the esophagus, intraepithelial papillary capillary loops and subepithelial capillary networks were identified. In the upper part of the stomach, polygonal subepithelial capillary loops surrounding the pits were observed, while in the lower part, surface epithelium with ridge‐like structures and coiled vessels were observed. A honeycomb pit structure and surrounding vascular structures were identified in the colon. Quantitative analysis showed the various contrasts of a single continuous vessel in the endoscopic image were due to different depths at which the vessel tortuously ran.

**Conclusion:**

We established a procedure to allow one‐to‐one correspondence between magnifying endoscopic and 3D reconstructed images and to measure the depth and caliber of endoscopically visualized vessels of interest. This method is expected to improve endoscopic diagnosis and further the development of endoscopic imaging technologies.

## INTRODUCTION

Image‐enhanced endoscopy is a medical optical procedure including various imaging technologies, such as narrow‐band imaging (NBI) or blue laser imaging, to enhance visualization during endoscopic procedures.^1^, ^2^ Magnifying endoscopy with NBI (ME‐NBI) enables the detailed visualization of superficial tissues by enhancing specific features such as microvessels and mucosal surface patterns.^3^, ^4^, ^5^ ME‐NBI has recently made it possible to predict the histology and estimate the invasion depth of gastrointestinal tract cancers, especially esophageal and colorectal cancers.^6^, ^7^ However, how changes in deeper tissues affect mucosal surface patterns is not fully understood. Clarifying the relationship between the deep tissue structure and the surface pattern will help to further our understanding of the morphological basis of this endoscopic diagnostic method. Histological examination is commonly used to visualize deeper tissue structures, but it only provides information in 2D vertical cross‐sections. To fully understand the relationship between deep tissue and surface structures, interpretation must be supplemented by the observer's imagination. Although 3D reconstruction using serial slices can resolve this problem, it is labor‐intensive and often results in distorted images^8^ as a result of tissue extension when mounted on glass slides.

Tissue‐clearing methods, which chemically homogenize the refractive index within tissue to reduce light scattering and make the tissue transparent, have recently been applied to visualize 3D tissue structures without slicing.^9^, ^10^ Using tissue‐clearing technology, some studies have reported 3D analyses of blood vessel and crypt structures in the digestive tract.^11^, ^12^ However, there are no reports of a procedure that can accurately correlate the mucosal surface in endoscopic images with the deeper tissue structures on a one‐to‐one basis for quantitative evaluation. By targeting a single blood vessel and tracing its continuity from the deeper to the mucosal surface, it becomes possible to accurately understand the nature of the blood vessel pattern. This approach allows accurate understanding of blood vessel patterns, which is important for endoscopic diagnoses, including those in the Japan Esophageal Society and the Japan NBI Expert Team classifications. The aim of this study was to develop a procedure for establishing a precise one‐to‐one correspondence between ME‐NBI images and 3D structures that can be quantitatively analyzed to clarify the relationship between surface patterns seen in endoscopic images and underlying deep tissue structures, with a focus on vessels in gastrointestinal neoplasms.

## METHODS

### Human specimens

This study involved a retrospective analysis of stored tissue specimens and endoscopic images that were collected between December 2018 and October 2022 at Showa University Northern Yokohama Hospital. Esophageal and gastric tissues were obtained by endoscopic submucosal dissection, and colon tissues were obtained by endoscopic mucosal resection. All tissue specimens were fixed in 10% buffered formalin and embedded in paraffin. The analyzed specimens included three esophageal squamous cell carcinomas, three gastric adenocarcinomas, one gastric adenoma, four colon adenomas, two sessile serrated lesions, and one colon adenocarcinoma. Detailed information on each specimen is presented in Table 1. Endoscopic images were captured using an EVIS X1 endoscopy system (Olympus Medical Systems, Tokyo, Japan) equipped with GIF‐XZ1200 or CF‐XZ1200 (both Olympus Medical Systems). The magnification power ranged from 70× to 80× for the esophagus and stomach and from 70× to 100× for the colon.

**Table 1:** Detailed information on each case and results of one‐to‐one correspondence

Case	Organ	Age (years)	Sex	Location	Shape	Histology	One‐to‐one correspondence
1	Esophagus	78	M	Mt	0‐IIc	Squamous cell carcinoma, T1a‐LPM	✓
2	Esophagus	75	M	Mt	0‐IIc	Squamous cell carcinoma, T1a‐LPM	✓
3	Esophagus	62	M	Ut	0‐IIc	Squamous cell carcinoma, T1a‐MM	✓
4	Stomach	76	M	U‐Ant	0‐IIc	Adenocarcinoma of the adenoma T1a(M)	✓
5	Stomach	83	M	L‐Less	0‐IIa	Adenoma, intestinal type	✓
6	Stomach	83	F	L‐Post	0‐IIa	Tubular adenocarcinoma, T1a(M)	✗
7	Stomach	61	F	U‐Ant	0‐IIa	Adenocarcinoma of the stomach, T1a(M)	✗
8	Colon	79	M	A	0‐Isp	Tubular adenoma, low grade	✓
9	Colon	73	F	S	0‐IIa	Tubular adenoma, low grade	✓
10	Colon	67	F	S	0‐IIa	Tubular adenoma, low to high grade	✓
11	Colon	59	M	S	0‐IIa	Sessile serrated lesion	✗
12	Colon	67	M	A	0‐IIa	Tubular adenoma low grade	✓
13	Colon	43	F	A	0‐IIa	Sessile serrated lesion	✗
14	Colon	73	M	Rs	0‐Is	Adenocarcinoma in adenoma, pTia	✓

✓, matched; ✗, not matched; A, ascending colon; F, female; L‐Less, lower third‐lesser curvature; L‐Post, lower third‐posterior wall; M, male; Mt, middle thoracic esophagus; Rs, rectosigmoid colon; S, sigmoid colon; U‐Ant, upper third‐anterior wall; Ut, upper thoracic esophagus.

### Tissue‐block selection

**Figure 1:**
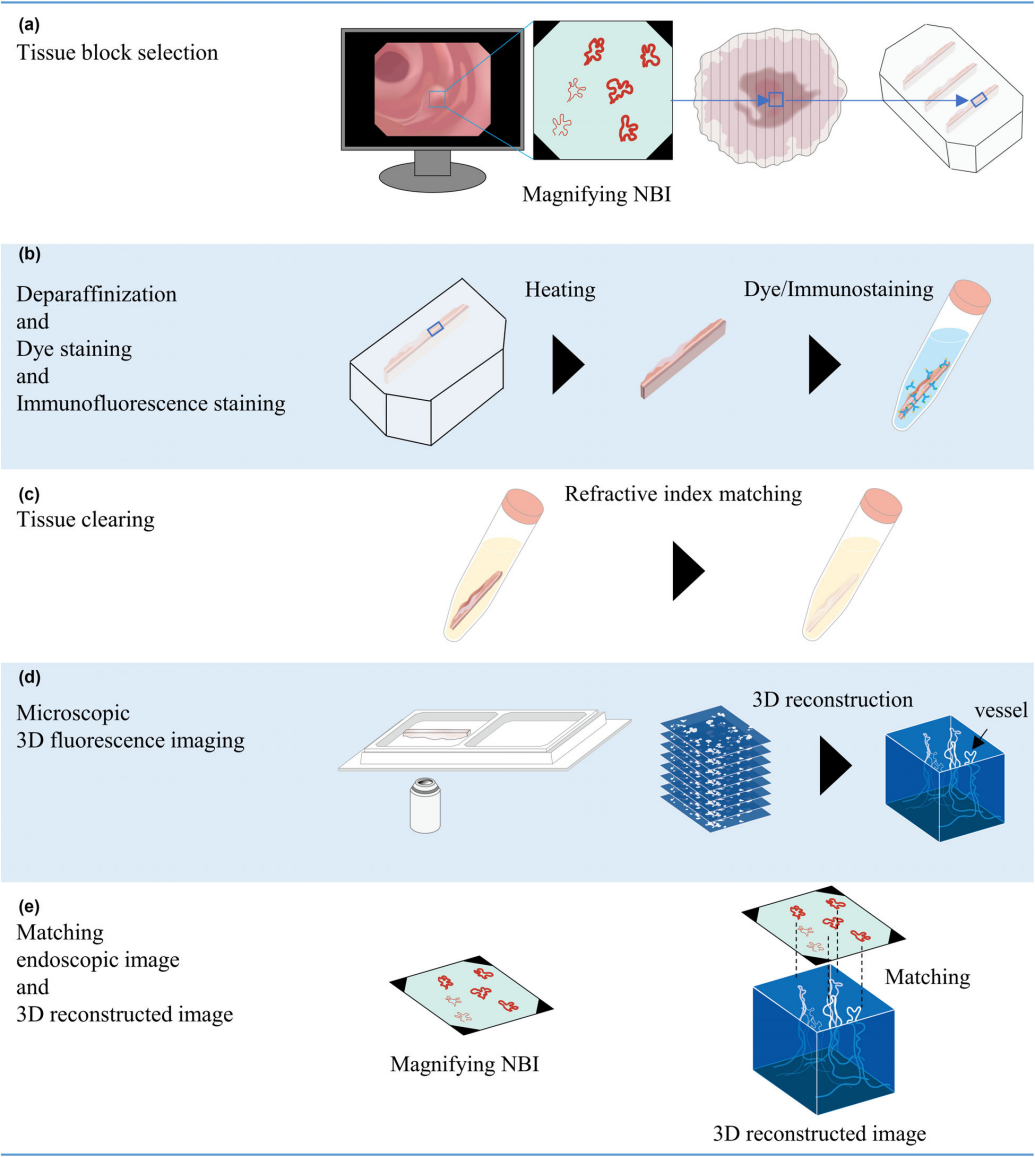
Overview of matching procedure between magnifying endoscopy with narrow‐band imaging (ME‐NBI) and 3D reconstructed images. (a) Identification of ME‐NBI observation site in resected tissue and selected paraffin block. (b) Deparaffinization and dye staining/immunostaining process. (c) Buffer change and refractive index matching. (d) Obtaining 3D fluorescence images using inverted confocal laser scanning microscopy. (e) Matching ME‐NBI and 3D reconstructed images using several crypt or vessel patterns as guides.

Specimens for which all three types of endoscopic images (white‐light imaging, NBI, and ME‐NBI) and stereomicroscopic images of resected material were available and were initially selected. The endoscopist and pathologist then selected one tissue block containing the endoscopic region of interest (ROI) from among multiply divided resected specimens, based on the endoscopic and resected tissue images. After selection, histological slides were made from the blocks for comparison with the 3D reconstructed images. For histological examination, tissue blocks were serially sectioned and stained with hematoxylin and eosin and immunostained with anti‐CD31 antibody (clone: JC70; Roche Diagnostics, Rotkreuz, Switzerland) and antipodoplanin antibody (clone: D2‐40; Roche Diagnostics). The blocks were then processed for whole‐tissue immunostaining and tissue clearing (Fig. 1). Further details are given in the methods in Appendix S1.

### Tissue‐block processing for creation of 3D reconstructed images

Tissue blocks were processed as described previously,^13^, ^14^ with some modifications (Fig. 1). Detailed information is provided in Table 2. Paraffin‐embedded tissues were deparaffinized and whole tissues were subjected to antigen retrieval and immunofluorescence staining with Alexa 647‐CD34 monoclonal antibody (clone: QBend10, FAB7227R; R&D Systems, Minneapolis, MN, USA) at 4°C for 14 days. For nuclear staining, SYTO16 (S7578; Invitrogen, Carlsbad, CA, USA) was added 7 days after the start of antibody incubation. Immunostained and optically cleared tissues were observed using an inverted confocal laser scanning microscopy system (FV3000; Evident, Tokyo, Japan). Optical‐section images were reconstructed in 3D using Imaris software (Bitplane AG, Zurich, Switzerland), and the 3D reconstructed images were basically viewed from two directions: a top‐view from a direction parallel to the mucosal plane, corresponding to the endoscopic observation, and a side‐view from a vertical cross‐sectional direction, corresponding to the histological plane.

**Table 2:** Procedure for establishing one‐to‐one correspondence between magnifying endoscopy with narrow‐band imaging and 3D reconstructed images

Procedure	Procedure details	Temperature	Incubation time	Day
Selection of tissue	Select the block‐embedded tissue containing the magnifying observation site within the lesion			
Deparaffinization	Cut out from paraffin‐embedded tissue block	RT	5 min	1
	Heating paraffin‐embedded tissue block on a paraffin block trimmer	62°C	5 min	
	Xylene	60°C	90 min	
	Xylene	60°C	90 min	
	100% ethanol	RT	60 min	
	70% ethanol	RT	60 min	
	50% ethanol	RT	60 min	
	Phosphate‐buffered saline	RT	Overnight	
Immunostaining	Antigen retrieval (pH 6 citrate buffer)	95°C	30 min	2–17
	Permeabilization	4°C	Overnight	
	Fluorescence immunostaining (CD34‐AlexaFluor647, dilution 1/100)	4°C	14 days	
	Fluorescence nucleic acid staining (SYTO16, dilution 1/1000)	4°C	7 days	
Tissue clearing	Phosphate‐buffered saline	4°C	6 h	18–20
	50% ethanol	4°C	12 h	
	70% ethanol	4°C	12 h	
	100% ethanol	4°C	12 h	
	Ethyl cinnamate	RT	1 day	
Fluorescence imaging	Confocal laser scanning microscopy (FV3000)	RT	1–2 h	21
Matching endoscopic and 3D reconstructed images	Matching endoscopic and 3D reconstructed images using crypts and vessels as a guide			

RT, room temperature.

### One‐to‐one correspondence and matching criteria

Three nonmedical engineers selected matching areas between the ME‐NBI and 3D reconstructed images by assessing similarities in the shapes of blood vessels and crypts and the relative positions of these structures. The selected matches were then confirmed by both a pathologist and an endoscopist, with only cases where both experts agreed being considered valid matches. The matching criteria required a minimum of five structures—either vessels alone or a combination of vessels and crypts—to exhibit the same shape and relative positions in both the ME‐NBI image and the 3D reconstructed image. For the esophagus, at least five vessels were required, while for the colon and stomach, a combination of vessels and crypts totaling at least five was needed for a valid match. If even one person out of the five observers did not agree, it was considered an unsuccessful match.

### Measurement of vessel depth and caliber in 3D reconstructed images

In cases where one‐to‐one correspondence was achieved, the depth of the vessels from the mucosal surface and vessel caliber were measured in side‐view images using Imaris (Bitplane) or ImageJ software (National Institutes of Health, Bethesda, MD, USA). To clarify the cause of differences in blood vessel visibility, ROIs were selected as areas where blood vessels exhibited varying appearances in ME‐NBI images.

## RESULTS

### 3D reconstructed images of optically cleared noncancerous gastrointestinal tissues

**Figure 2:**
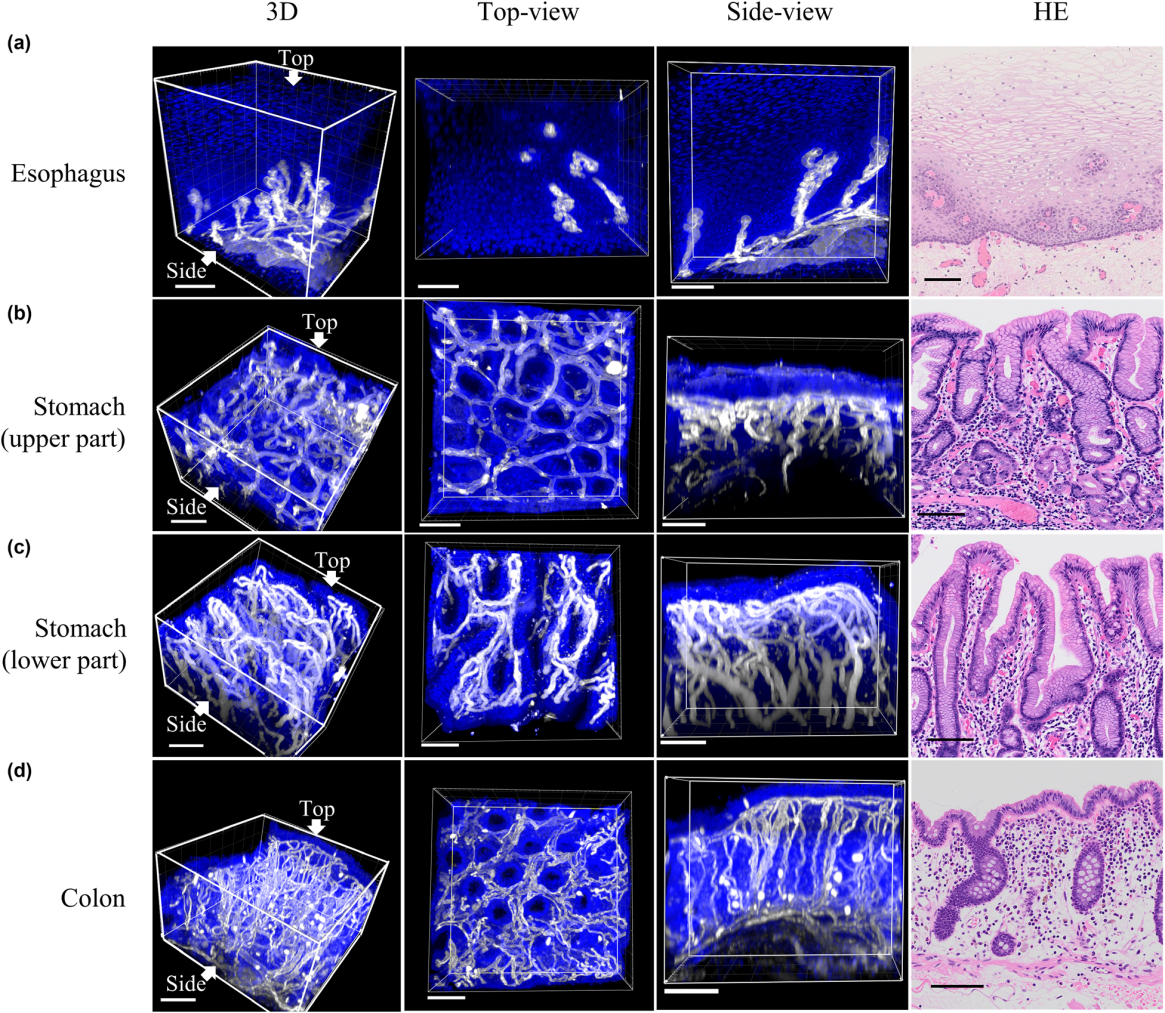
3D reconstructed images of noncancerous gastrointestinal tissues. 3D reconstructed images of (a) esophagus, (b) upper part of stomach, (c) lower part of stomach, and (d) colon. Nuclei stained with SYTO16 (blue) and blood vessels immunostained with anti‐CD34 antibody (white). Hematoxylin and eosin (HE) images showing corresponding tissue histology. Top‐view from mucosal surface displays a thickness of 250 μm (esophagus) and 150 μm (stomach, colon); side‐view from vertical section displays a thickness of 150 μm. Scale bar: 100 μm (white, black).

All paraffin‐embedded tissues from the esophagus, stomach, and colon were optically cleared with refractive index matching (Fig. S1). This enabled all epithelial and mesenchymal cell nuclei and blood vessel structures to be visualized in all layers of endoscopically resected tissue, including both tumor and adjacent nontumor tissues, using a 10× objective lens (Fig. S2a). Images of nuclei and blood vessels were captured to a depth of ~350 μm in optically cleared tissues using a 30× objective lens (Fig. 2). Intraepithelial papillary capillary loops and the subepithelial capillary network were clearly visualized in the esophagus in 3D imaging (Fig. 2a, Video S1). The caliber of the intraepithelial papillary capillary loops near the mucosal surface was ~10 μm, and the caliber of the drainage vein in the proper mucosal layer was ~50 μm (Fig. 2a). In the upper part of the stomach, 3D vessel imaging showed a polygonal subepithelial capillary loop surrounding the pit, with each capillary forming part of a regularly arranged network (Fig. 2b, Video S2). In the lower part of the stomach, the surface epithelium of the ridge‐like structure was observed, and the capillary morphology was coiled (Fig. 2c, Video S3). Images of microvessels arranged in a honeycomb pattern around the crypts were obtained in the colon (Fig. 2d, Video S4). The 3D vessel images showed ascending and descending microvessels of different calibers.

### 3D visualization of blood vessel network in neoplastic gastrointestinal tissues

**Figure 3:**
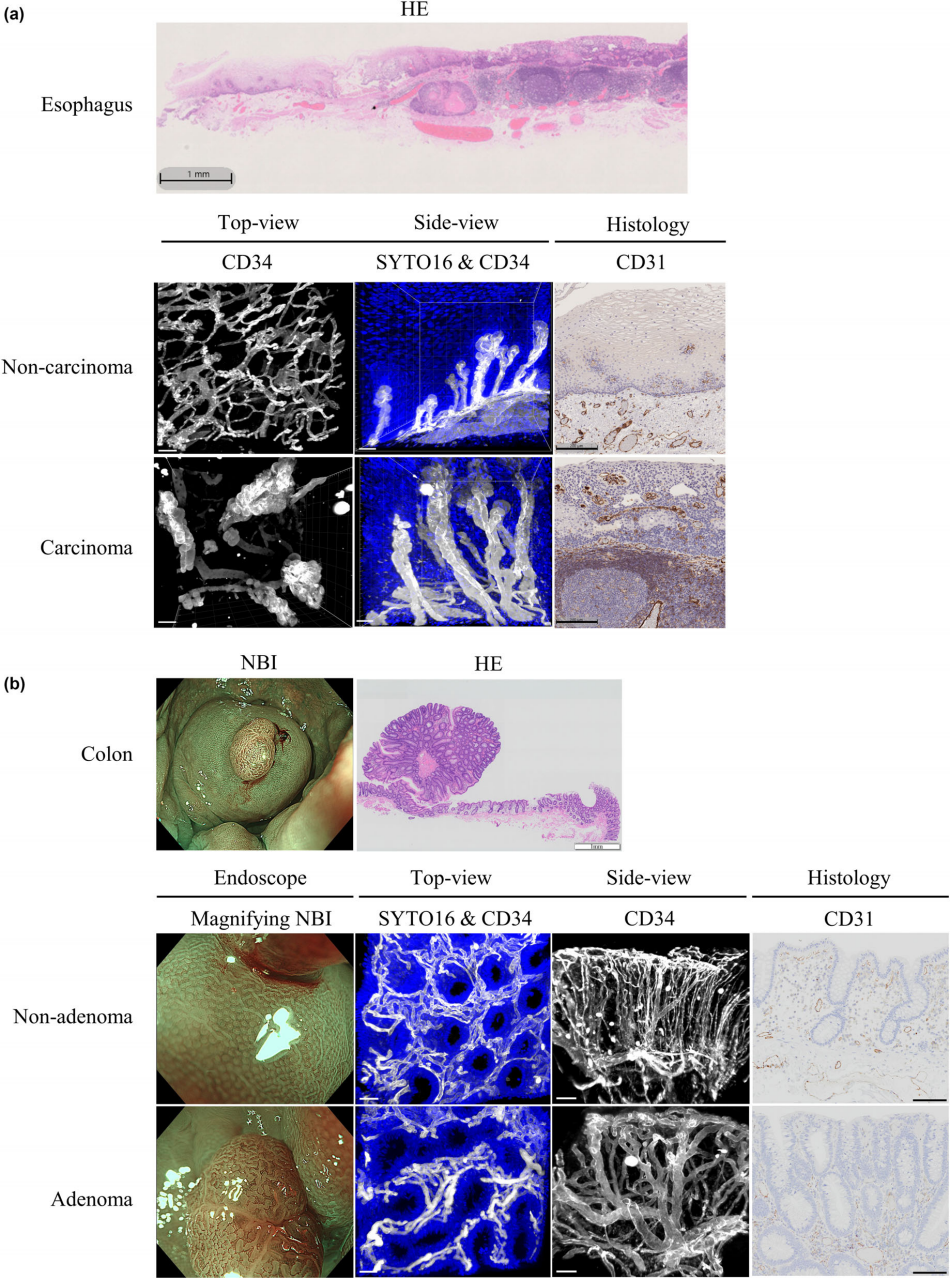
3D reconstructed images of cancerous gastrointestinal tissues. 3D reconstructed images of (a) esophagus and (b) colon and identical endoscopic images. Nuclei stained with SYTO16 (blue) and blood vessels immunostained with anti‐CD34 antibody (white). Hematoxylin and eosin (HE) images show corresponding tissue histology. (a) Top‐view image shows blood vessels only; side‐view image shows nuclei and blood vessels in noncarcinoma and carcinoma tissues. CD31‐immunostained tissue slides of same region shown. Scale bars: 50 μm (white), 200 μm (black). (b) Identical magnifying endoscopic images with narrow‐band imaging (NBI) and top‐view images show nuclei and blood vessels; side‐view images show blood vessels only. CD31‐immunostained tissue slides of the same region shown. Scale bars: 50 μm (white), 100 μm (black).

In the esophagus, 3D visualization showed that looped vessels in the intraepithelial component of cancer tissues were significantly more dilated and tortuous than those in noncancerous tissues (Fig. 3a). In addition, the side‐view showed that the vessel height was significantly greater in cancerous than noncancerous tissues (Fig. 3a), and the difference was statistically confirmed (*P* < 0.05) (Fig. S2b,c).

In colon adenoma, the top‐view showed that the crypt orifice was oval and nonuniform in size, while that in nonadenoma tissues was circular and uniform in size. These features corresponded to the ME‐NBI images (Fig. 3b). In the side‐view, intralesional microvessels were more dilated and significantly more tortuous in adenoma than nonadenoma areas (Fig. 3b). This difference was also statistically significant (*P* < 0.05) (Fig. S3).

### One‐to‐one correspondence of identical vessels and/or crypt structure of gastrointestinal tissues between ME‐NBI and 3D reconstructed images

**Figure 4:**
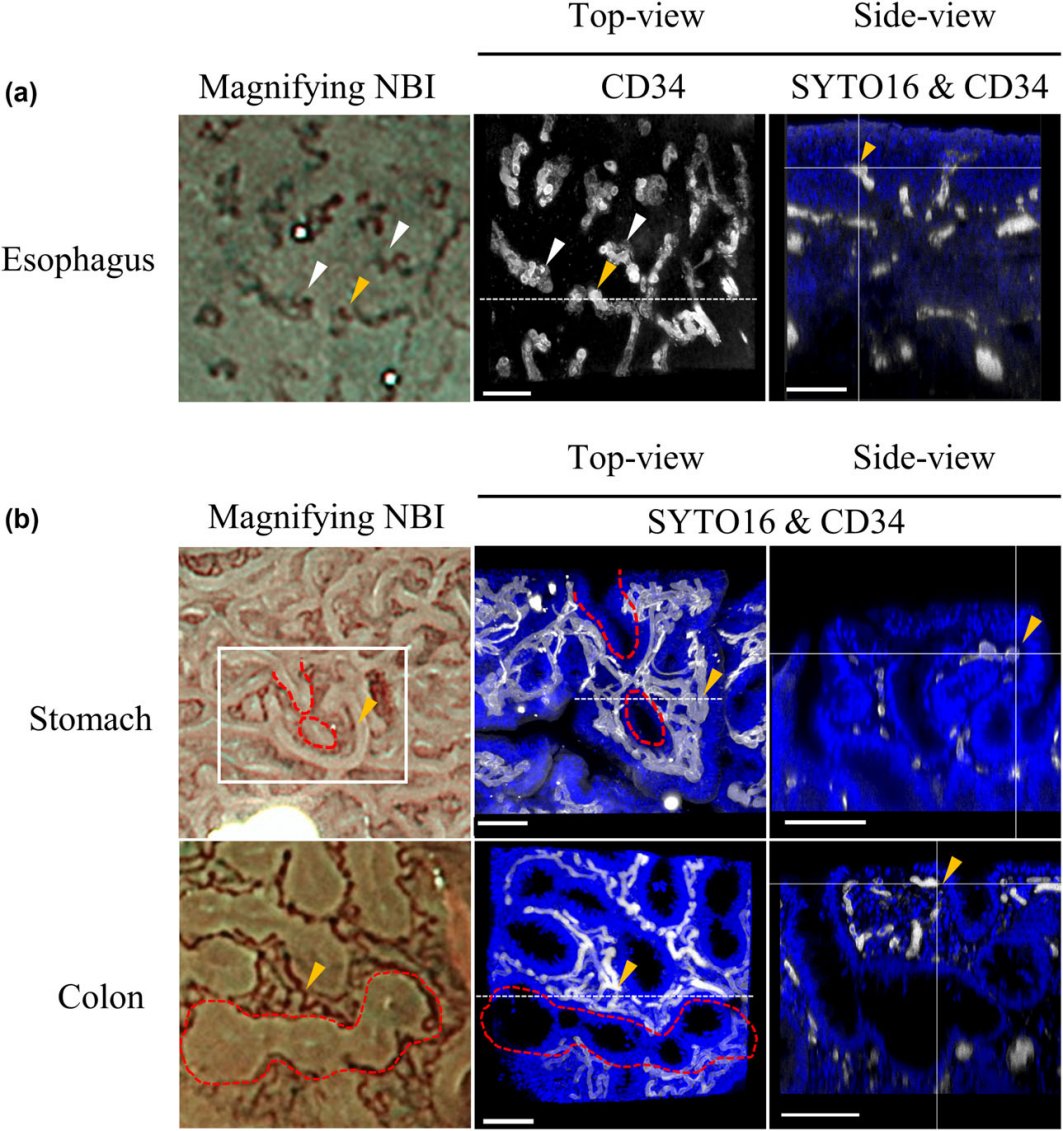
One‐to‐one correspondence between magnifying endoscopy with narrow‐band imaging (ME‐NBI) and 3D reconstructed images of vessels in gastrointestinal tissues. 3D reconstructed images of (a) esophagus and (b) stomach and colon. Nuclei stained with SYTO16 (blue) and blood vessels immunostained with anti‐CD34 antibody (white). (a) Top‐view image of blood vessels with yellow and white arrowheads pointing to the same vessels as those shown in ME‐NBI image; side‐view (white dashed section in left panel) shows vertical cross‐section of vessel indicated by yellow arrowhead in left panel. (b) Yellow arrowhead indicates blood vessel, and area enclosed by red dotted line contains crypts. Top‐view shows nuclei and blood vessels with yellow arrowhead pointing to same vessel as shown in ME‐NBI image; side‐view shows vertical cross‐section of vessel indicated by yellow arrowhead. Scale bar: 100 μm (white).

**Figure 5:**
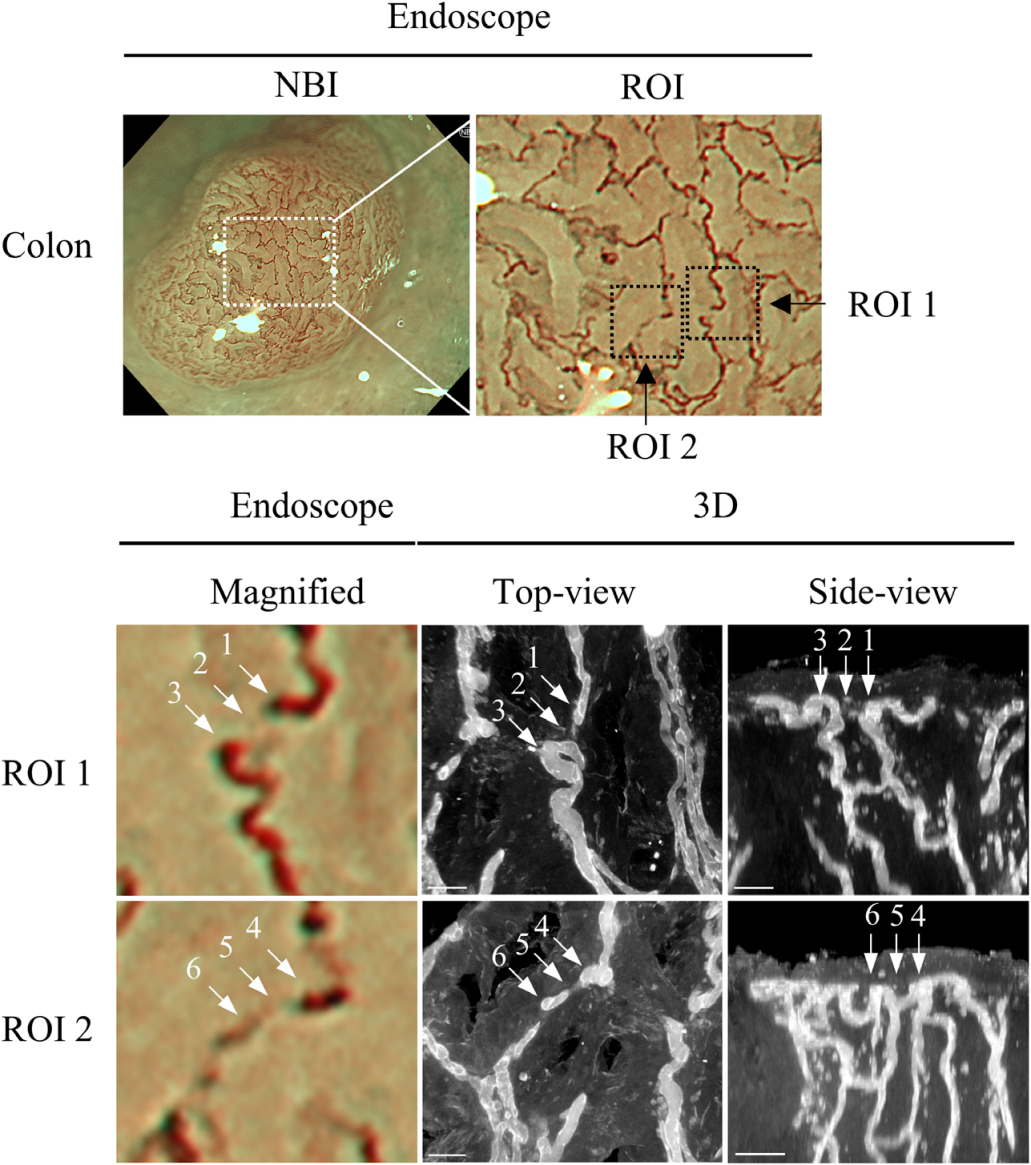
Measurement of vessel depth and caliber in the region of interest (ROI) in the colon, focusing on vessels with varying visibility. Numbered vessels within lesions are indicated by white arrows in endoscopic images. Top‐view and side‐view images of blood vessels (CD34) correspond to numbered vessels in colon. Scale bar: 50 μm (white). NBI, narrow‐band imaging.

The one‐to‐one corresponding microvessel structures in ME‐NBI images were successfully identified in 3D reconstructed images for all three cases of esophageal squamous cell carcinoma (Fig. 4a, Table 1), while correspondence was demonstrated for two of the four gastric lesions and five of the seven colorectal lesions (Fig. 4b,c, Table 1). In cases where one‐to‐one correspondence was successfully established, the depth and caliber of blood vessels observed via endoscopy could be measured (Figs S4,S5, Table S1). In the colon, this method enabled detailed analysis of histological characteristics, such as changes in vascular contrast relative to the background mucosa, focusing on the relationship between vessel caliber and depth by targeting a single continuous blood vessel (Fig. 5). In vessel no. 1–3, the blood vessels appeared disconnected under magnified endoscopy; however, in the 3D tissue image, these vessels were confirmed to extend to a depth of >100 μm without interruption. In vessel no. 4–6, the vascular contrast changed when the blood vessel path shifted at a depth of 20 μm (Fig. 5, Table 3). Regarding the measurements, the depth from the mucosal surface and caliber of the blood vessels using the tissue‐clearing method were correlated with those in the histological slides (Fig. S6).

**Table 3:** Vessel depth from mucosal surface and caliber of colon adenoma

Case	Vessel no.	3D	
		Depth (μm)	Caliber (μm)
No. 9 colon: tubular adenoma	1	28.0	10.0
	2	108.9	6.2
	3	20.3	15.1
	4	35.0	12.1
	5	54.6	11.1
	6	32.3	11.1

## DISCUSSION

The 3D images obtained by the herein‐described method were consistent with the anatomical knowledge established by scanning electron microscopy and corrosion casts.^15^, ^16^ The precise 3D reconstructed images captured nuclei and blood vessels up to a depth of ~350 μm from the surface using a 30× objective lens, which covered the depth reached by endoscopic imaging.^17^ This suggests that this method may be suitable for enhancing the understanding of endoscopic images.

This successful one‐to‐one correspondence analysis enabled quantitative measurements of the target vessels' caliber and depth from the mucosal surface as observed in endoscopic images. Moreover, the measurement results for depth and vessel visibility were similar to those of previous reports that analyzed cases of colorectal adenomas and sessile serrated lesions using tissue specimens.^18^ These data suggest that our method provides reasonable measurements of blood vessels. When developing imaging technology, it is essential to accurately correlate endoscopic images with quantitative data on blood vessel caliber and depth. The differences in the visibility of the same blood vessel caused by different wavelengths, such as NBI and red dichromatic imaging, can be quantitatively evaluated using this method.

This study had several limitations. First, one‐to‐one correspondence may not be achievable in all cases. The main reasons for failure included the inability to identify characteristic vessels, such as large blood vessels, in the NBI images and the relatively uniform surface structure of the lesion, which lacked clear landmarks for matching.

Second, when acquiring high‐resolution images with a 30× objective lens, the observable area is limited to a small region. Although the resolution is reduced, the use of a 10× objective lens makes it possible to observe deep structures such as blood vessels that are visible with red dichromatic imaging.

Third, because this study focuses on methodology, the number of cases analyzed was limited. Consequently, a comprehensive comparison of the magnifying endoscopic classification of each organ and the 3D reconstructed images could not be performed. However, we believe that future studies involving a sufficient number of well‐selected representative cases with crucial endoscopic findings will provide valuable insights. Such studies could enhance the understanding of categories that are challenging to estimate for depth of invasion using the current magnifying endoscopy classifications, such as B2 vessels in the Japan Esophageal Society Classification and Type 2B lesions in the Japan NBI Expert Team Classification.

In conclusion, we developed a nondestructive 3D analysis method for formalin‐fixed paraffin‐embedded gastrointestinal normal and neoplastic tissues using tissue clearing and fluorescence immunostaining. We confirmed that quantitative analysis could be carried out for vessels showing one‐to‐one correspondence between ME‐NBI and 3D reconstructed images. This method is expected to not only improve endoscopic diagnosis, but also further the development of endoscopic imaging technologies.

## FUNDING INFORMATION

This work was supported by Olympus Medical Systems Corp.

## CONFLICT OF INTEREST

Authors K.N., K.M., S.M., N.O., and I.S. are employees of Olympus Medical Systems Corp., which supported this work. M.M. and S.K. received speaking honoraria from Olympus Marketing Corp. M.M. is an Associate Editor of *Digestive Endoscopy*. The other authors declare no conflict of interest for this article.

## ETHICS STATEMENT

Approval of the research protocol by an Institutional Review Board: This study was approved by the Institutional Review Board of Showa University Northern Yokohama Hospital (ethical approval no. 20H115) and Olympus Corporation (ethical approval no. OLET‐2020‐010).

Informed Consent: All samples were obtained by the opt‐out method, in accordance with the Helsinki Declaration. All participants were enrolled between 1 January 2005 and 30 June 2023 at Showa University Northern Yokohama Hospital.

Registry and Registration No. of the Study/Trial: N/A.

Animal Studies: N/A.

## Supporting information


**Figure S1** Refractive index matching with ethyl cinnamate. Representative bright‐field images of gastrointestinal tissues and cleared using ethyl cinnamate. Grid size: 5 mm.

**Figure S2** Quantitative comparison of vessel height between cancerous and noncancerous esophageal tissues. (a) Hematoxylin and eosin (HE) image and corresponding 3D (3D) reconstructed image of esophageal tissue; nuclei stained with SYTO16 (blue), and blood vessels immunostained with anti‐CD34 antibody (white). Scale bar: 500 μm. Orange dotted rectangle, cancerous region; blue dotted rectangle, noncancerous region. Scale bar: 200 μm. (b) Representative 3D height of vessel in cancerous region. (c) Difference in vessel height between cancer and noncancerous regions (*n* = 10).

**Figure S3** Quantitative comparison of vessel tortuosity between adenoma and nonadenoma colon tissues. (a) Illustrations and mathematical formulas showing the definition of tortuosity. (b) Difference in tortuosity between adenoma and nonadenoma regions (*n* = 3). Further details are given in the Appendix S1.

**Figure S4** Measurement of vessel depth from mucosal surface and vessel caliber in the region of interest (ROI) in esophagus. (a) Endoscopic image of esophagus and ROI numbers within lesion indicated by white arrows. (b) Top‐view and side‐view images of blood vessels corresponding to ROI numbers. Scale bar: 100 μm. NBI, narrow‐band imaging.

**Figure S5** Measurement of vessel depth from mucosal surface and vessel caliber in the region of interest (ROI) in the stomach. (a) Endoscopic image of stomach and ROI numbers within lesion indicated by white arrows. (b) Top‐view and side‐view images of blood vessels corresponding to ROI numbers. Scale bar: 100 μm. NBI, narrow‐band imaging.

**Figure S6** Quantitative comparison of 3D reconstructed images and histology of vessel depth within lesions in the colon. (a) Vessel depth in 3D images and histology of adenomas and sessile serrated lesions. (b) Correlation coefficient between average vessel depths in 3D and histological images. (c) Image of vessel depth by 3D reconstruction and histology.

**Appendix S1** Supporting methods.

**Table S1** Vessel depth from mucosal surface and caliber of esophagus and stomach.

**Video S1** Three‐dimensionally reconstructed image of esophagus.

**Video S2** Three‐dimensionally reconstructed image of upper part of stomach.

**Video S3** Three‐dimensionally reconstructed image of lower part of stomach.

**Video S4** Three‐dimensionally reconstructed image of colon.

## Data Availability

The data that support the findings of this study are available from the corresponding author upon reasonable request.
