# Supplementary figures and images for: Three‐dimensional optically cleared tissue imaging for analyzing endoscopic images of gastrointestinal neoplasms (with video)
Source: Dig Endosc. 2025 Feb 3;37(6):659–69. doi: 10.1111/den.15000 (PMC12162411; doi:10.1111/den.15000)

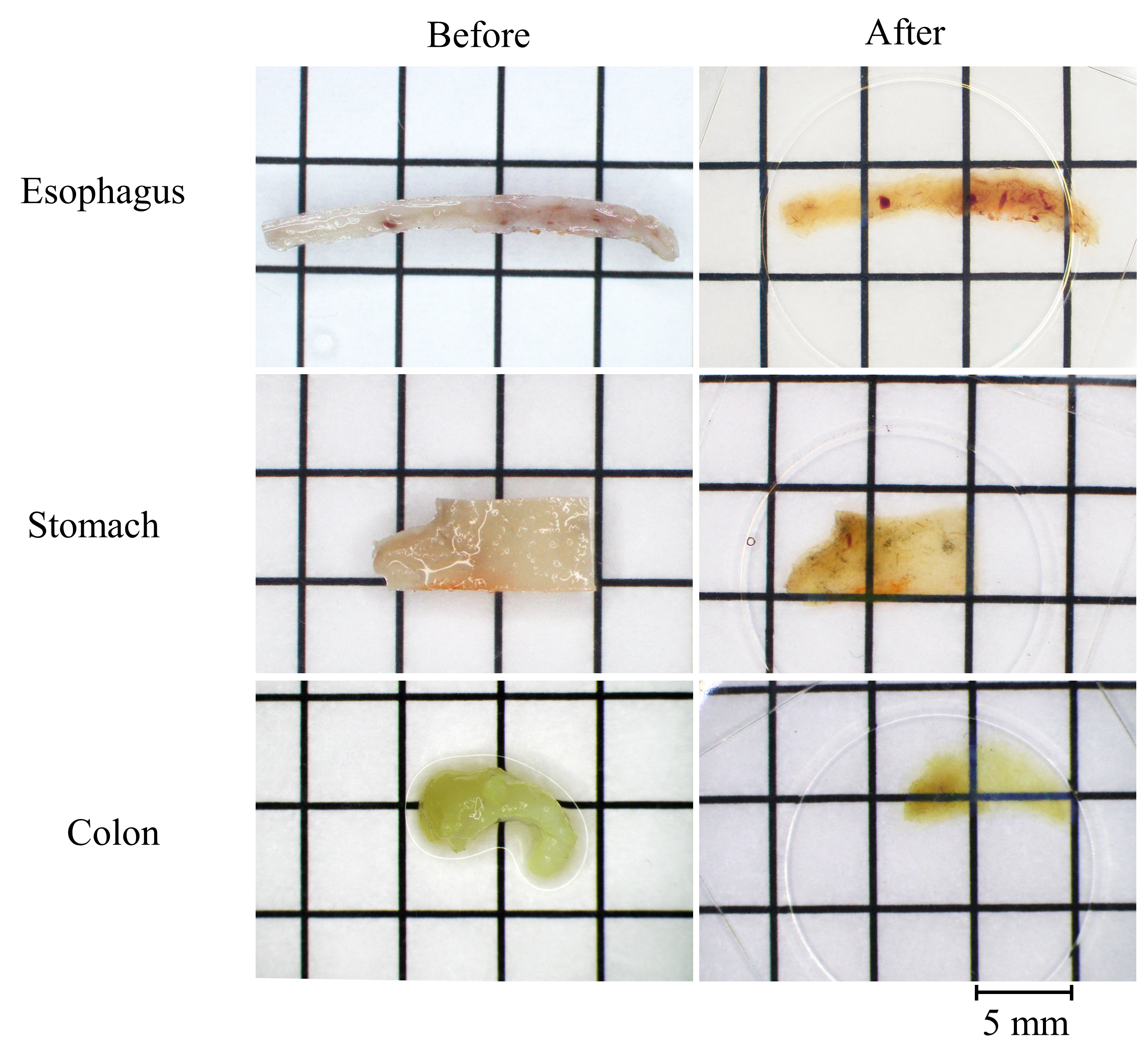

Supplement: Supplementary file 1 — Figure S1 Refractive index matching with ethyl cinnamate. Representative bright‐field images of gastrointestinal tissues and cleared using ethyl cinnamate. Grid size: 5 mm. [file DEN-37-659-s007.tif]

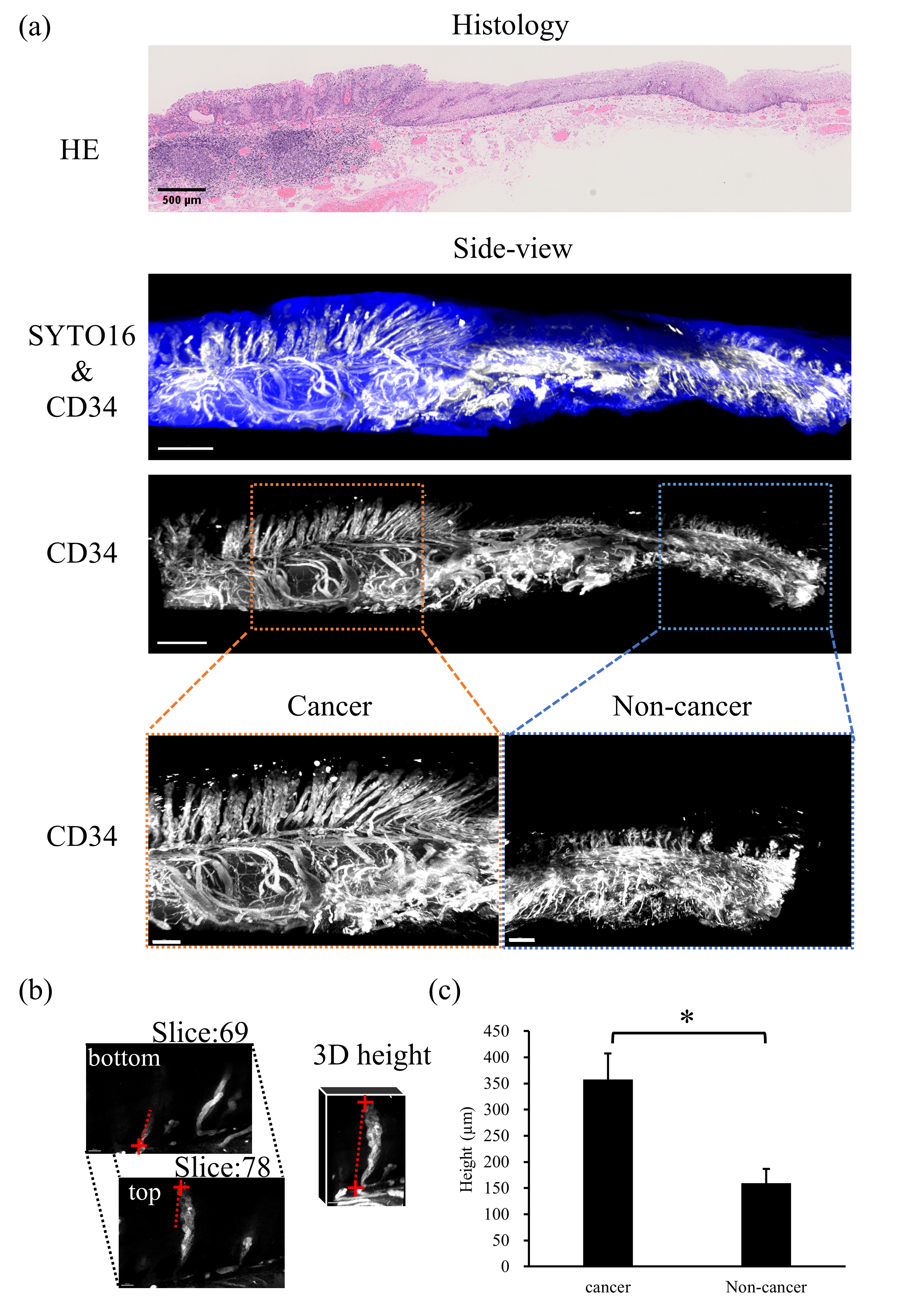

Supplement: Supplementary file 2 — Figure S2 Quantitative comparison of vessel height between cancerous and noncancerous esophageal tissues. (a) Hematoxylin and eosin (HE) image and corresponding 3D (3D) reconstructed image of esophageal tissue; nuclei stained with SYTO16 (blue), and blood vessels immunostained with anti‐CD34 antibody (white). Scale bar: 500 μm. Orange dotted rectangle, cancerous region; blue dotted rectangle, noncancerous region. Scale bar: 200 μm. (b) Representative 3D height of vessel in cancerous region. (c) Difference in vessel height between cancer and noncancerous regions (n = 10). [file DEN-37-659-s001.tif]

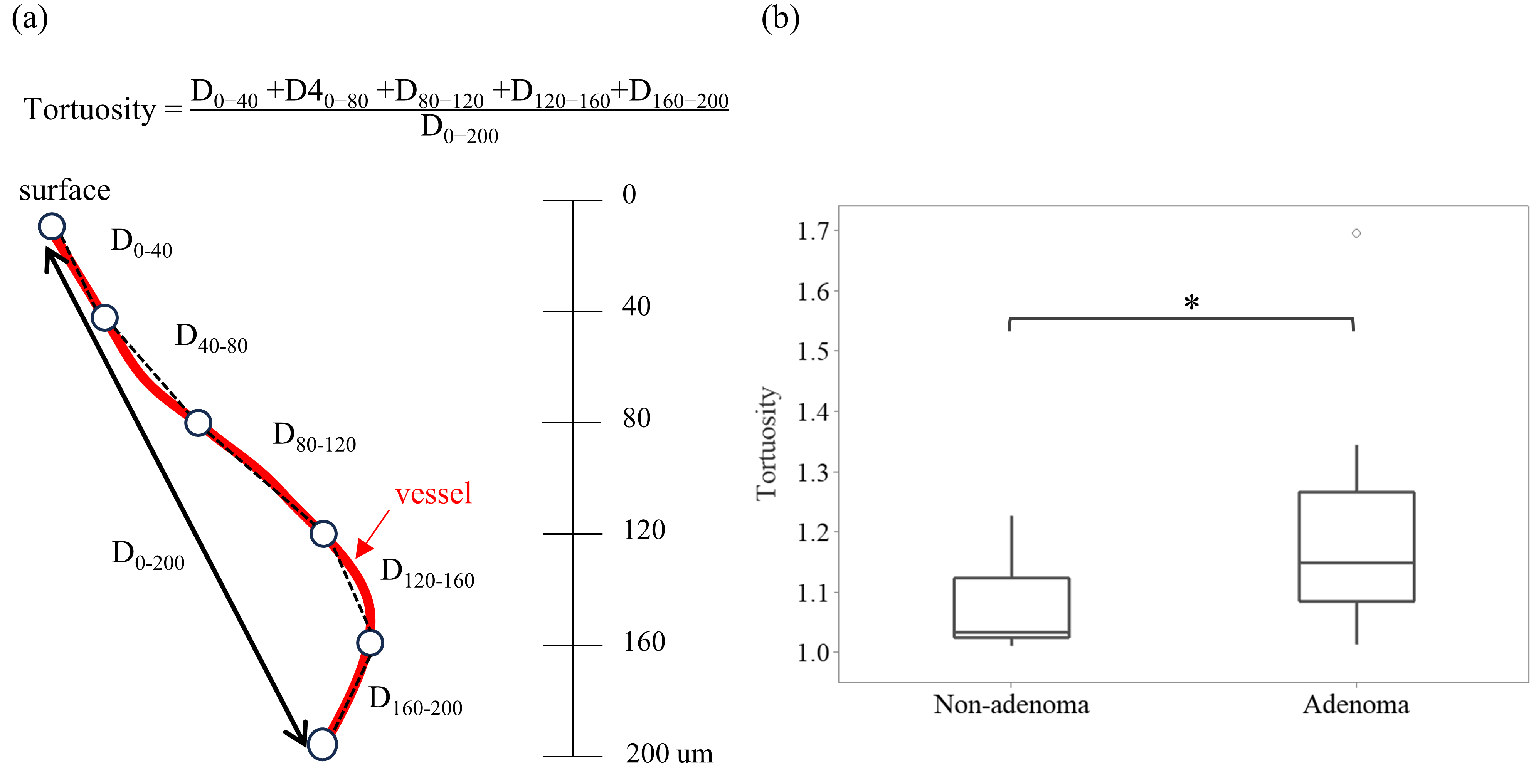

Supplement: Supplementary file 3 — Figure S3 Quantitative comparison of vessel tortuosity between adenoma and nonadenoma colon tissues. (a) Illustrations and mathematical formulas showing the definition of tortuosity. (b) Difference in tortuosity between adenoma and nonadenoma regions (n = 3). Further details are given in the Appendix S1. [file DEN-37-659-s009.tif]

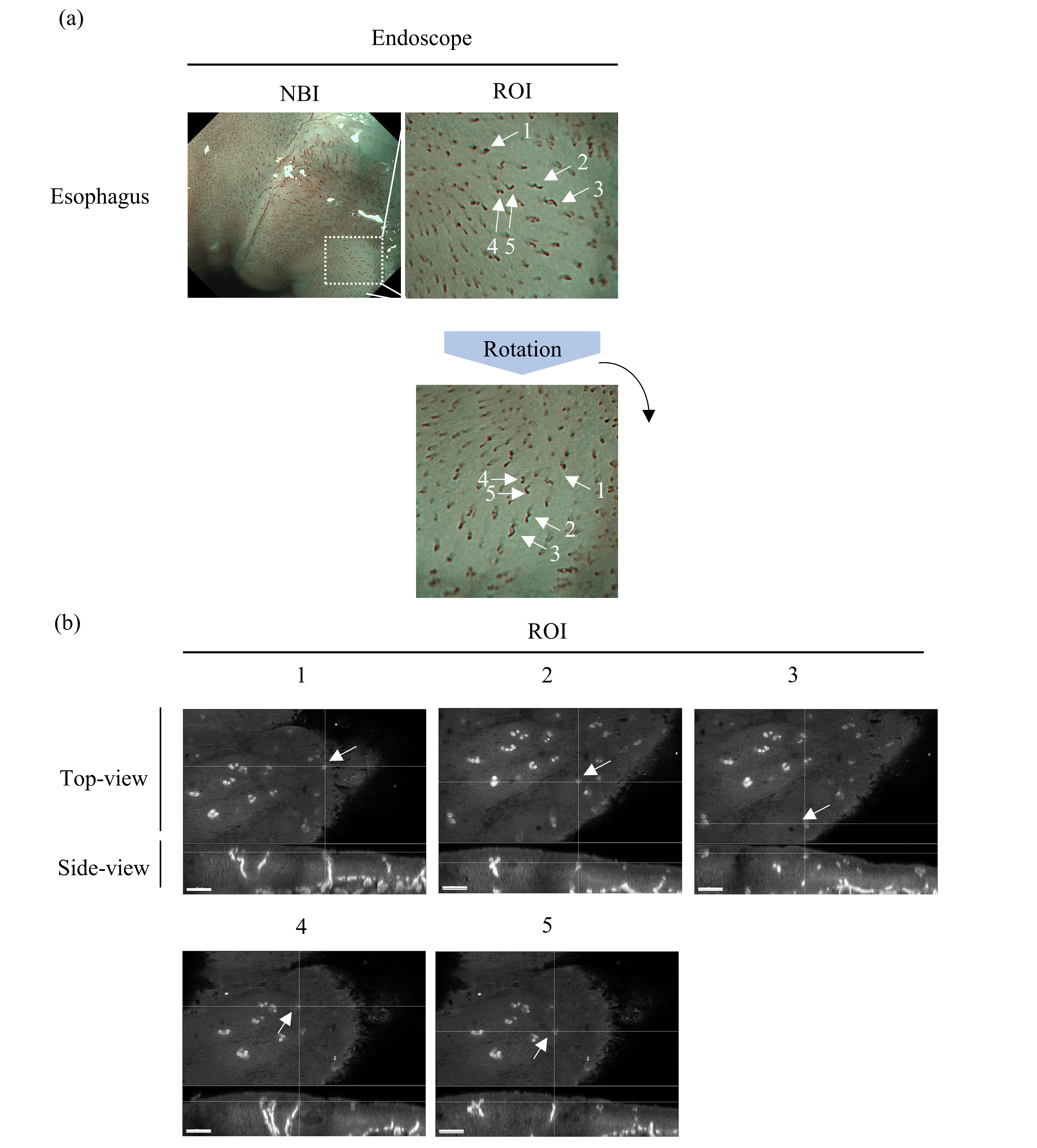

Supplement: Supplementary file 4 — Figure S4 Measurement of vessel depth from mucosal surface and vessel caliber in the region of interest (ROI) in esophagus. (a) Endoscopic image of esophagus and ROI numbers within lesion indicated by white arrows. (b) Top‐view and side‐view images of blood vessels corresponding to ROI numbers. Scale bar: 100 μm. NBI, narrow‐band imaging. [file DEN-37-659-s012.tif]

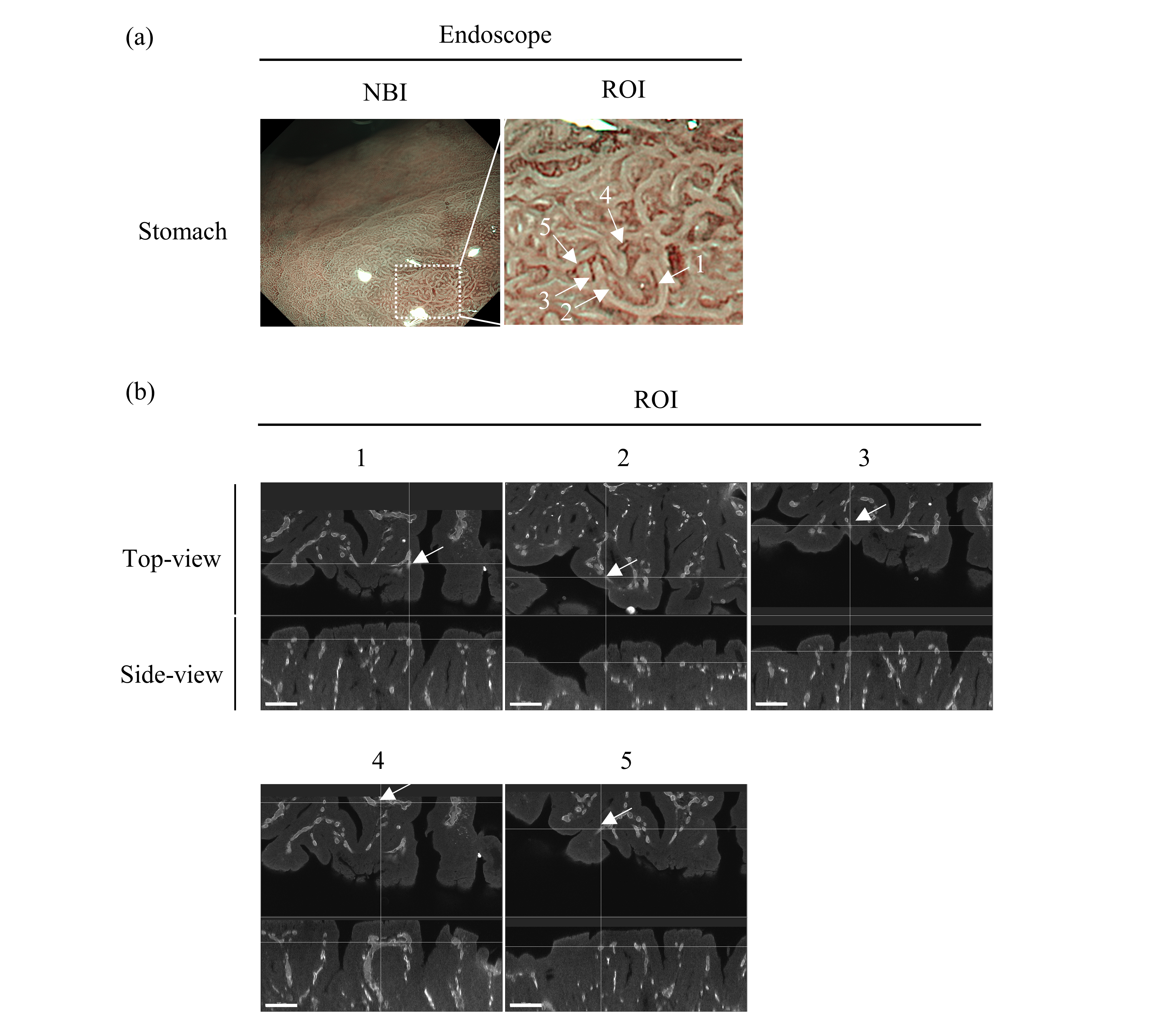

Supplement: Supplementary file 5 — Figure S5 Measurement of vessel depth from mucosal surface and vessel caliber in the region of interest (ROI) in the stomach. (a) Endoscopic image of stomach and ROI numbers within lesion indicated by white arrows. (b) Top‐view and side‐view images of blood vessels corresponding to ROI numbers. Scale bar: 100 μm. NBI, narrow‐band imaging. [file DEN-37-659-s006.tif]

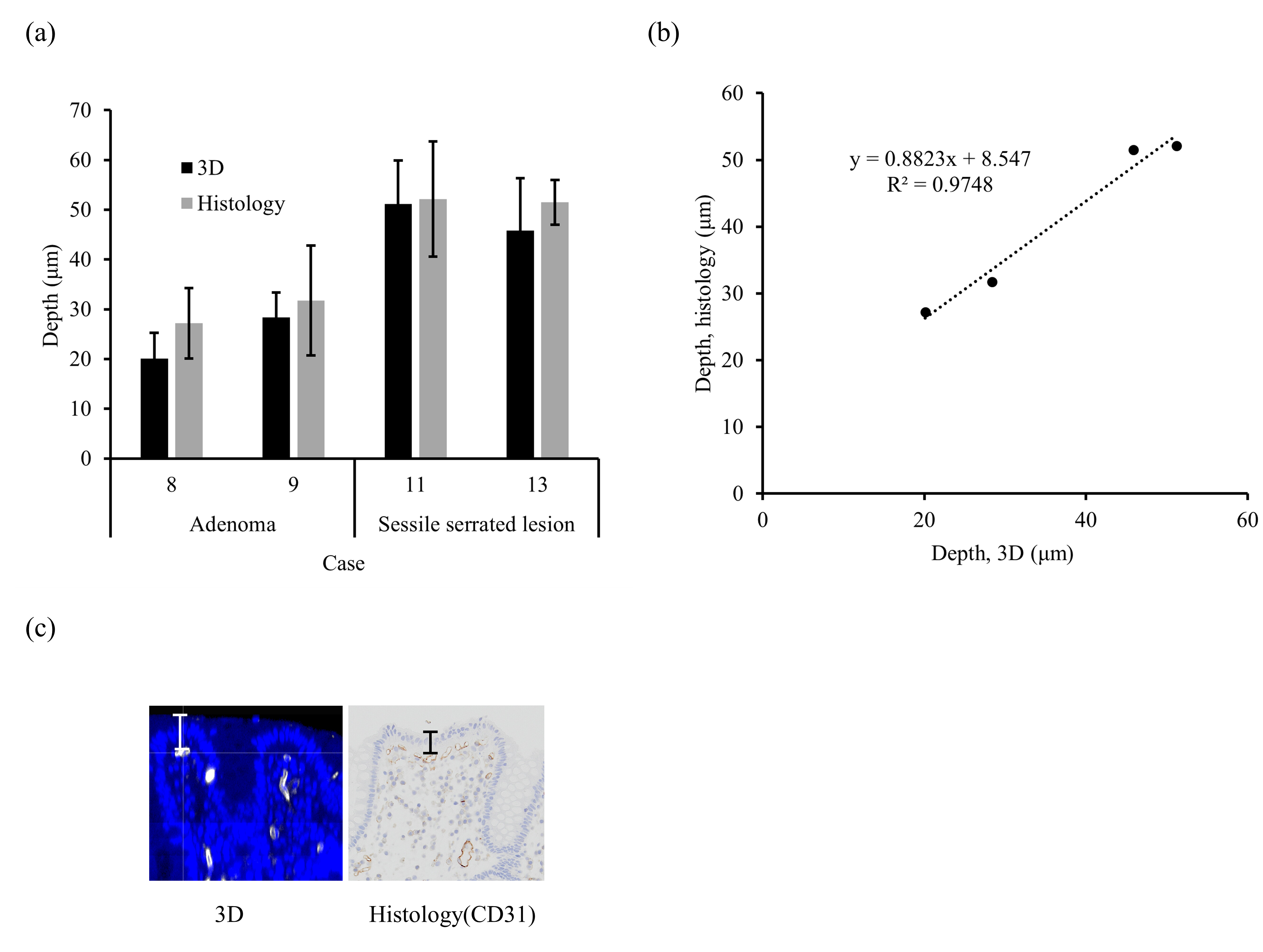

Supplement: Supplementary file 6 — Figure S6 Quantitative comparison of 3D reconstructed images and histology of vessel depth within lesions in the colon. (a) Vessel depth in 3D images and histology of adenomas and sessile serrated lesions. (b) Correlation coefficient between average vessel depths in 3D and histological images. (c) Image of vessel depth by 3D reconstruction and histology. [file DEN-37-659-s002.tif]
