# Supplementary material for: Three‐dimensional optically cleared tissue imaging for analyzing endoscopic images of gastrointestinal neoplasms (with video)
Source: Dig Endosc. 2025 Feb 3;37(6):659–69. doi: 10.1111/den.15000 (PMC12162411; doi:10.1111/den.15000)
Supplement: Supplementary file 7 — Appendix S1 Supporting methods. [file DEN-37-659-s011.docx]

**Supporting Methods**

**Imaging and analysis of tissue sections**

Tissue section images were captured using a whole-slide imaging system (VS200; Evident Corporation., Tokyo, Japan) equipped with a 20× objective lens (UPLXAPO20x; Evident Corporation). Tissue section images were subjected to measurement analysis using VS200 ASW software (Evident Corporation).

**Tissue-block processing for 3D reconstructed images**

Paraffin-embedded tissues were trimmed using a blade to remove excess paraffin, deparaffinized, and subjected to antigen retrieval in heated retrieval solution (Dako Target Retrieval Solution Citrate pH 6, S2369; Agilent, CA, USA), followed by incubation at 4°C overnight in a permeabilization and blocking solution (Phosphate Buffered Saline (T900, Takara Bio, Shiga, Japan) + 0.25% Triton X-100 (T9284, Sigma-Aldrich, MO, USA) + 1% Bovine Serum Albumin (013-27054, FUJIFILM Wako, Osaka, Japan)). Whole-tissue immunofluorescence staining was performed by incubating with Alexa 647-CD34 monoclonal antibody (dilution: 1/100, clone: QBend10, FAB7227R; R&D Systems, CA, USA) at 4°C for 14 days. Nuclei were stained by adding SYTO16 (dilution: 1/1000, S7578; Invitrogen, MA, USA) 7 days after the initiation of antibody incubation. Tissue clearing was performed by 24-h incubation with ethyl cinnamate (112372, Sigma-Aldrich) at room temperature with gentle shaking under dark conditions (Table 1).

**Imaging and analysis of 3D reconstructed images**

Immunostained and optically cleared tissues were observed using an inverted confocal laser scanning microscopy system (FV3000; Evident Corporation) equipped with a 10× objective lens (UPLSAPO 10x; Evident Corporation) and a 30× silicone immersion objective lens (UPLSAPO30x SIR, Evident Corporation). Optical-section images were captured with a z-step size of 4.2 µm for the 10× objective lens and 0.8 µm for the 30× objective lens. For nuclear imaging, the SYTO16 fluorescence signal was excited by a 488 nm laser and detected at 500–540 nm with a variable barrier filter. For microvessel imaging, the Alexa647-CD34 fluorescence signal was excited by a 640 nm laser and detected at 650–750 nm with a variable barrier filter.

**Measurement of tortuosity**

Measurements were taken from the mucosal surface to a depth of 200 μm, and blood vessels were traced from the surface to the deep layer and marked at intervals of 40 μm. The starting point (D_0_) of measurement was defined as the point where the superficial blood vessels branch into the deeper layers. The sum of the distances of each marking point and the shortest distance from the top of the blood vessel to a depth of 200 μm (D_0_- D_200_) were measured. The degree of tortuosity was calculated using the ratio of these distances.

**Statistical analysis**

The mean (± standard deviation) heights of vessels in non-cancerous and cancerous tissues in the esophagus, and the mean tortuosity of vessel in non-adenoma and adenoma tissue in the colon, were determined using a two-sample *t*-test with Minitab Software (version 21.4.2; PA, USA), with a level of significance of p < 0.05.
