# Supplementary material for: Three‐dimensional optically cleared tissue imaging for analyzing endoscopic images of gastrointestinal neoplasms (with video)
Source: Dig Endosc. 2025 Feb 3;37(6):659–69. doi: 10.1111/den.15000 (PMC12162411; doi:10.1111/den.15000)
Supplement: Supplementary file 8 — Table S1 Vessel depth from mucosal surface and caliber of esophagus and stomach. [file DEN-37-659-s005.docx]

| **Case** | **Vessel** | **3D** | |
| --- | --- | --- | --- |
|  | **No.** | **Depth (µm)** | **Caliber (µm)** |
| No.2  Esophagus: squamous cell carcinoma | 1 | 14.1 | 18.7 |
|  | 2 | 22.7 | 24.5 |
|  | 3 | 35.3 | 12.5 |
|  | 4 | 31.1 | 10.5 |
|  | 5 | 22.7 | 10.5 |
| No.5  Stomach: adenoma | 1 | 49.6 | 11.9 |
|  | 2 | 52.9 | 10.5 |
|  | 3 | 45.5 | 11.7 |
|  | 4 | 69.0 | 12.0 |
|  | 5 | 47.1 | 8.6 |

**Table S1** Vessel depth from mucosal surface and caliber of esophagus and stomach
